# Focus on the morphogenesis, fate and the role in tumor progression of multivesicular bodies

**DOI:** 10.1186/s12964-020-00619-5

**Published:** 2020-08-08

**Authors:** Xueqiang Peng, Liang Yang, Yingbo Ma, Yan Li, Hangyu Li

**Affiliations:** Department of General Surgery, The Fourth Affiliated Hospital, China Medical University, Shenyang, 110032 China

**Keywords:** Multivesicular body, Extracellular vesicles, Amphisome, Autophagy, Trafficking, Release, Cancer

## Abstract

Multivesicular bodies (MVBs) are endosome organelles that are gradually attracting research attention. Initially, MVBs were considered as important components of the endosomal-lysosomal degradation pathway. In recent years, with an increase in extracellular vesicle (EV) research, the biogenesis, fate, and pathological effects of MVBs have been increasingly studied. However, the mechanisms by which MVBs are sorted to the lysosome and plasma membrane remain unclear. In addition, whether the trafficking of MVBs can determine whether exosomes are released from cells, the factors are involved in cargo loading and regulating the fate of MVBs, and the roles that MVBs play in the development of disease are unknown. Consequently, this review focuses on the mechanism of MVB biogenesis, intraluminal vesicle formation, sorting of different cargoes, and regulation of their fate. We also discuss the mechanisms of emerging amphisome-dependent secretion and degradation. In addition, we highlight the contributions of MVBs to the heterogeneity of EVs, and their important roles in cancer. Thus, we attempt to unravel the various functions of MVBs in the cell and their multiple roles in tumor progression.

**Video Abstract**

**Video Abstract**

## Background

Multivesicular bodies (MVBs) are organelles defined by a single membrane, which typically have a diameter of about 250 nm to 1000 nm, and contain smaller 50–80 nm diameter intraluminal vesicles (ILVs). Morphologically, most MVBs are round or slightly elliptical [1–5]. They were first discovered in the nervous system in the 1950s, and the “gold standard” definition is based on their ultrastructural morphology [3, 4]. Altick et al. systematically elaborated the distribution, protein content, and trafficking function of MVBs in neurons, and further revealed the classification, function, and properties of MVBs in the hypoglossal nerve [3, 4]. Notably, the discovery of MVBs has expanded from the original nervous system to the entire life domains, including mammals, plants, fungi, and other organisms, and they are highly conserved in both yeast and mammalian systems [3, 6, 7]. The typical roles of MVB is to participate in protein trafficking in the endocytic system and to regulate homeostasis of the endosomal-lysosomal pathway [2, 8]. However, as research has progressed, the focus has shifted to the cell microenvironment, leading to the discovery that the microenvironment contains an abundance of meaningful extracellular vesicles (EVs), which can significantly change the behavior of target cells, especially in the tumor microenvironment [9–12]. Importantly, the fusion of MVBs with the plasma membrane then releases ILVs into the extracellular space as exosomes [13–15].

The contents of mature MVBs are generally divided two broad categories: constitutive molecules and cargo molecules [3, 4]. The constitutive molecules are essential for the organelle function, constituting the organelle structure or serving MVBs functions, which includes endocytic processes, vesicle budding, sorting functions, and numerous signaling molecules involved in the regulation of MVB fate [16, 17]; for example, endosomal sorting complex required for transport (ESCRT), ceramide, tetraspanin proteins (CD81, CD9, CD37,and CD63), small GTPases (Rabs), and synaptosomal-associated proteins (SNAREs) [18, 19]. On the one hand, the cargo molecules might comprise factors derived from MVB sorting, including processed and transported membrane-bound receptors, ligands, internalized proteins, and macromolecules. On the other hand, the cargoes may comprise active proteins, nucleic acids, lipids, and/or substances recruited from the cytoplasm [2, 20–23]. Different subpopulations of MVBs consist of different constituent molecules, and exert different physiological and pathological effects [21, 23–25]. Unfortunately, there have been few studies on the variations in MVB morphology and distribution. The distribution and content level of MVBs show significant heterogeneity in a variety of cells and pathological processes [2, 3, 26, 27]. In this review, we systematically summarize the origin of MVBs, and the mechanisms of cargo sorting and MVB fate regulation. We also discuss the contributions of MVBs to the heterogeneity of EVs. Finally, the effects of MVBs on cancer progression are summarized.

Not surprisingly, it seems that only a comprehensive disclosure of MVB involved in the endosomal-lysosomal system degradation and the selectivity of diverse cargoes loading of MVB for discharging their ILVs into the extracellular space as exosomes mechanisms [14, 28–30]. We can have a more multifaceted understanding of the role of MVBs in cancer growth and metastasis to provide new ideas for further manipulation of MVB fates [9, 24].

### MVB biogenesis

**Fig. 1:**
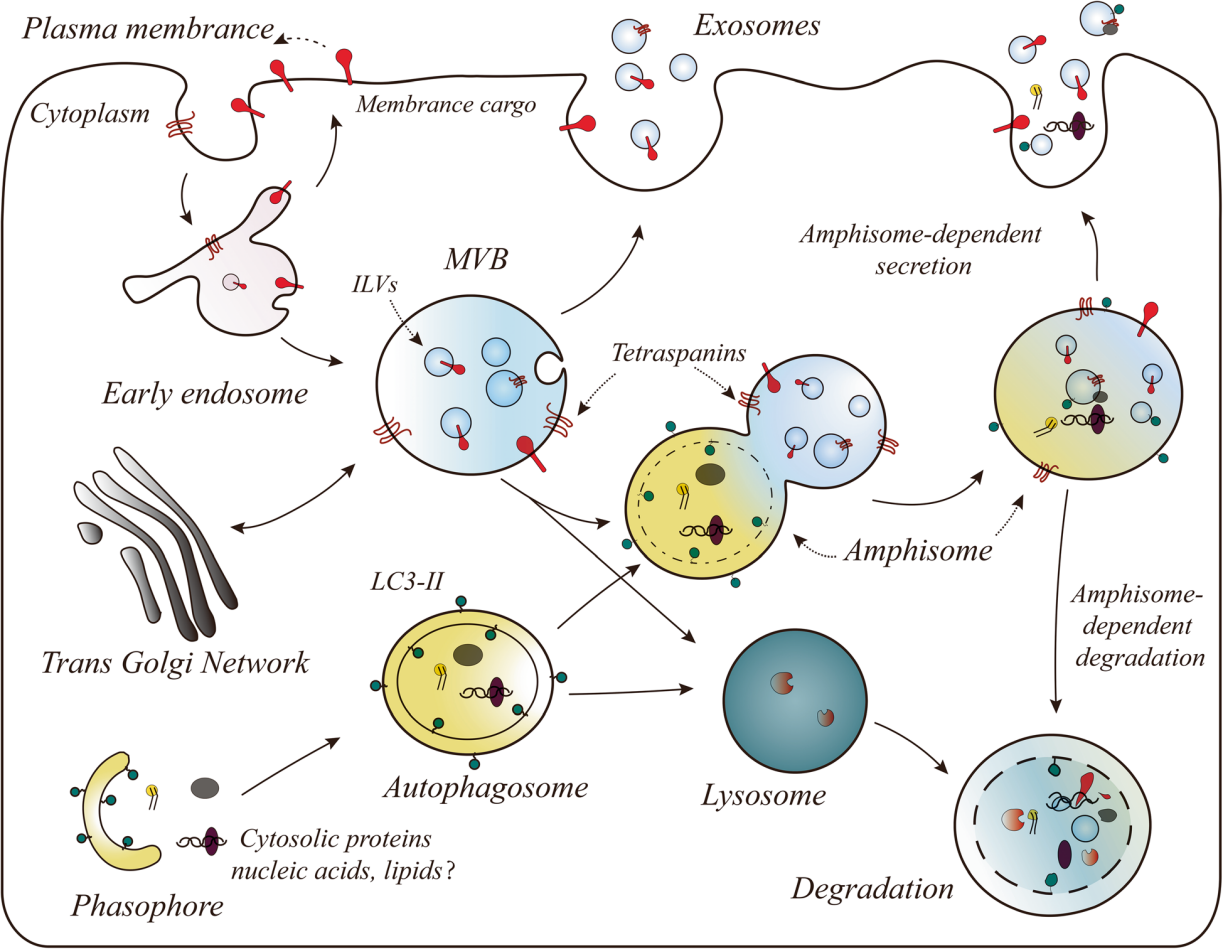
(Multivesicular body) MVB morphogenesis and possible sorting pathways: exosome release, back-fusion, and degradation in the lysosome and amphisome-dependent degradation or secretion. (1) the MVB may fuse with the plasma membrane and release the ILVs as exosomes. (2) Membrane cargo (ligand/receptor) may be recycled back to the plasma membrane or may be targeted to ILVs in the MVB. (3) MVBs can target internalized membrane cargoes (ligand/receptors) for degradation in the lysosome by fusing with lysosomes. (4) The amphisome fuses with lysosomes to form the autolysosome for degradation of cargo, or fuses with the plasma membrane, triggering extracellular component release, including dsDNA, proteins and lipids and separately, ILVs act as exosomes

Originally, it was believed that endocytosis and Golgi secretion were sorted into special early endosomes with different cargoes, and then, the early endosomes were thought to internalize and form ILVs under an endosomal sorting complex required for transport (ESCRT) to form mature MVBs [1, 31–33]. Misfolded proteins, signalling receptors, and related factors were thought to be sorted into MVBs and then degraded into lysosomes to maintain intracellular material balance and homeostasis [11, 22, 29, 33–35]. However, further in-depth research showed that eukaryotic MVBs serve as key components of the plasma membrane quality control system by identifying and degrading cell surface proteins and intracellular misfolded proteins [25, 33, 36–38]. Moreover, MVBs selectively load specialized substances (lipids, proteins, and nucleic acids) and then fuse with the plasma membrane to release their exosomes [13, 19, 39, 40] (Fig. 1). Importantly, how are mature MVBs formed? Studies have demonstrated that early endosomes in the endosomal system gradually generate ILVs under an ESCRT-dependent or ESCRT-independent mechanism, and the formation of mature MVBs is a critical first step in this process [31, 41, 42].

#### The ESCRT-dependent mechanism

MVB biogenesis requires mechanisms to create distinct domains along the endosomal membrane and further produces ILVs [2, 43, 44]. ESCRT complexes (ESCRT-0, −I, −II, and -III) and ESCRT-III-associated proteins (e.g., vacuolar protein sorting gene 4 (VPS4) and ALIX (disassembly complex)) have been further studied [2, 43]. Briefly, ALIX and ESCRT-I/ESCRT-II are directly involved in membrane budding, and ESCRT-III and the AAA-ATPase VPS4 subsequently cut the bud from the cytoplasm to form ILVs [43, 45, 46]. The specific functions and structure of the ESCRT subunits have been further studied [43, 47]. During the formation of ILVs, the coordinated binding of VPS4 to ESCRT-III drives vesicle neck contraction [31, 43]. The number of ILVs was significantly reduced in cells with double mutations of VPS2 and Snf7 (ESCRT-III), while showing a larger ILV neck, and the whole process was inseparable from the synergistic effect of VPS4 in yeast [31]. Interestingly, Wenzel et al. confirmed that one ESCRT wave results in the formation of a single ILV at a time via observing the number of ILVs at different time points after EGF stimulation correlated well with the number of waves observed from live-cell imaging at the corresponding time points [44]. In addition, they also revealed that clathrin not only plays a role in endocytosis, but is also located on the MVB membrane where it seems to interfere with HRS membrane localization, which further affects the formation of ILVs [44].

#### The ESCRT-independent mechanism

The biogenesis of ESCRT-independent ILVs involves a mechanism in which ceramide induces vesicle bending and budding [42, 48–50]. Activation of Gi-coupled sphingosine 1-phosphate (SP1) receptors on MVBs is essential for cargo sorting into the ILVs destined for exosome release [48]. Proteolipid protein is transferred to distinct subdomains on the endosomal membrane, and then exosome-associated domains are transferred into the lumen occurs in an ESCRT-independent manner, which also requires the sphingolipid ceramide [49]. Im et al. [50, 51] depleted the ESCRT subunits and also inhibited ceramide activity, confirming that sulfamethoxazole regulated ESCRT-dependent MVB biogenesis and secretion. In fact, the ceramide-mediated mechanism may be relatively independent of ESCRT-dependent ILV biogenesis; however, studies have shown that ESCRT-mediated cargo sorting can synergize with ceramide-induced lipid curvature to produce vesicles that share both ESCRT-dependent and -independent mechanisms [19, 49, 50]. Interestingly, other mechanisms for ILVs biosynthesis have been proposed [26, 52, 53]. Natural adiponectin has a multimeric structure, and adiponectin and T-cadherin play key roles in membrane filling, which can significantly induce ILV biogenesis [26]. In addition, adiponectin possibly enhances ILV formation via a mechanism that is independent of ceramide and ESCRT [26, 43]. Notably, syndecans, via their attached heparan sulfate polysaccharide chains, bind to their cytoplasmic junction syntenin to participate in the formation of ILVs [54]. Syntenin also interacts directly with ALIX through the three LYPX_n_L motifs, and in turn, ALIX can bind to ESCRT-III, thereby creating the machinery critical for cargo deposition into the ILVs of the MVBs [53–55]. Moreover, syntenin-ALIX exosome biogenesis and budding into MVBs can be controlled by ADP ribosylation factor 6 and phospholipase D2 [53]. Of interest, tetraspanins, including CD63, CD9, CD81, and CD82, comprise integral membrane proteins that are highly enriched in exosomes, [23, 41, 56]. Association with tetraspanins has emerged as completely independent of the ESCRT-dependent and ceramide sorting mechanisms for promoting entry into the MVBs [23, 56].

### Sorting of cytoplasmic cargoes into MVBs

Notably, active substances released in the cytoplasm might need to be recruited close to the MVB membrane to be sequestered into the ILVs [57, 58]. However, how these substances are sorted into MVBs remains largely unknown.

#### Protein cargoes

Post-translational modifications (PTMs) of proteins participate in the loading of specific elements into the ILVs of MVBs [58, 59]. In particular, ubiquitin and ubiquitin-like modifiers are major controllers of protein loading in MVBs [58, 59]. A recent study showed that ubiquitin-like 3 (UBL3) modification controls protein sorting into MVBs [58]. However, does a cytoplasmic protein need to be sorted into a specific membrane region via specific transporter recognition after PTMs [58]? Interestingly, a new mechanism has been identified for proteins harbouring a KFERQ motif, enabling their binding to heat shock protein 70 (HSP70), which is involved in the delivery of endosomal microautophagy (eMI)-dependent cytoplasmic proteins to MVBs in an ESCRT dependent manner [60, 61]. Furthermore, wnt induces the arginine-methylated proteins, glycogen synthase kinase 3 (GSK3) and protein arginine methyltransferase 1 to be encompassed within a membrane-bound organelle, which is also translocated into MVBs in an eMI-dependent manner [62]. Recent studies suggest that autophagic substrates (p62) can enter MVBs via the eMI pathway via a rapid starvation reaction that depends on ESCRT-III and VPS4 [63, 64]. In addition, another study revealed that a protein complex, made of two hydrolytic enzymes and the autophagic adaptor NBR1, is delivered into ILVs inside MVBs in the ubiquitination and ESCRT-dependent mechanism. This specific mechanism has been termed the NVT pathway (NBR1-mediated vacuolar targeting) [61, 65, 66]. Notably, the mechanisms of the eMI and NVT pathways respectively address the cytoplasmic proteins either in the lumen of the forming ILVs or in the lumen of the MVB outside of the ILVs [61, 66]. Obviously, it is worth noting that these mechanisms (eMI and NVT) of MVB formation/loading are more likely to target lysosomal pathway degradation processes, whereas the mechanism of the exosomal protein recruitment has not yet been completely characterized.

#### Nucleic acid cargoes

Notwithstanding, exosomal RNAs are able to modulate pathophysiological processes, and the mechanisms controlling specific RNA sorting into MVBs are just beginning to be understood [20, 67–71]. Based on RNA structures, accumulating evidence suggests that RNAs with the same motif sequences may be targeted to ILVs via RBPs (RNA-binding proteins) [13, 20, 68, 72]. For example, cytosolic Y-box protein 1 (YBX1) can recognize specific motifs mRNAs and selectively package these RNAs into ILVs [20]. Furthermore, heterogeneous nuclear ribonucleoprotein A2B1 (hnRNPA2B1) can derive the loading of exosomal microRNAs into MVBs by binding to specific Exo-motifs [73]. Major vault protein (MVP) can package miR-193a into exosomes leading to the reduction of cytoplasmic miR-193a via forming an MVP protein-miR-193a complex [72]. It has been reported that RISC, argonaute 2 (Ago2) and GW182 can be combined with packaged RNAs into ILVs [13, 67, 74]. Endosomal membranes are also involved in regulating the formation and turnover of the RISC complex, which is beneficial for RISC to continuously recruit target RNA into MVBs [13, 75]. Moreover, a study showed that the KRAS proto-oncogene GTPase (KRAS) inhibits the sorting of Ago2-dependent miRNAs into MVBs [67]. Weaver et al. further revealed the existence of KRAS-dependent sorting of multiple RNAs [74]. An additional mechanism for nucleic acid cargo sorting into ILVs involves the NURR (N-terminal unit for RNA recognition) domain of SYNCRIP (synaptotagmin-binding cytoplasmic RNA-interacting protein), which directly targets miRNAs containing hEXO motifs into ILVs [68]. In addition, Hobor et al. revealed that the molecular basis of Syncrip-mediated special RNA loading into ILVs, which was recognition of the miRNA targets is mediated by the cooperation between a NURR RNA-binding domain and three RNA recognition motifs (RRMs) in Syncrip [76]. However, the commonly reported exosomal RBPs examined (Ago, hnRNPA2B1, PARK7/DJ1, GAPDH, and MVP) were absent from classical exosomes when standard purification methods were performed, suggesting that potential RBPs and RNA isolation mechanisms or are necessary processes when RNA is loaded into MVBs [13].

Most researchers’ research on the mechanism of RNA loading into ILVs has focused on the recognition and recruitment of specific RNA motifs by RNA-binding proteins (see above). There are few studies on how RNA is sorted into specific regions of an MVB and then is incorporated into ILVs. Several studies have demonstrated that in transporting RNA to MVBs is probably dependent on the ability hnRNPA2B1 to travel along the cytoskeleton [73]. YBX1 may participate in miRNA loading into ILVs through ubiquitin modification and/or direct binding to components of the ESCRT mechanism or other ubiquitin-modified integral membrane proteins [43, 77]. Notably, transported by RBPs, RNA continuously interacts with the outer (cytoplasmic) surface of MVBs. Importantly, RNAs with higher affinity for raft-like regions of the MVB limiting membrane are retained at the membrane and then loaded into ILVs through different internalization mechanisms [71, 78]. Therefore, we can speculate that RNA molecules initially engage MVBs and then are incorporated into ILVs in a process that largely depends on the modification and regulation of the RBPs, specific RNA sequence motifs and affinity for membrane lipids. Surprisingly, many previous studies believed that microRNAs are mainly stable inside exosomes. A recent study by Rong Xu et al. [79] reported that a large number of exosomal miRNA species bound to RBPs can reside on the outer surface of the vesicle. Although there are few reports of microRNAs on the surface of exosomes, recent studies have revealed that microRNA may adhere to the outer membrane of exosomes. There is no doubt that the determination of microRNAs on the exosome surface will have an important impact will have on the study of RNA loading mechanisms and exosome-mediated miRNA cell-cell communication.

### Fates of MVBs

**Fig. 2:**
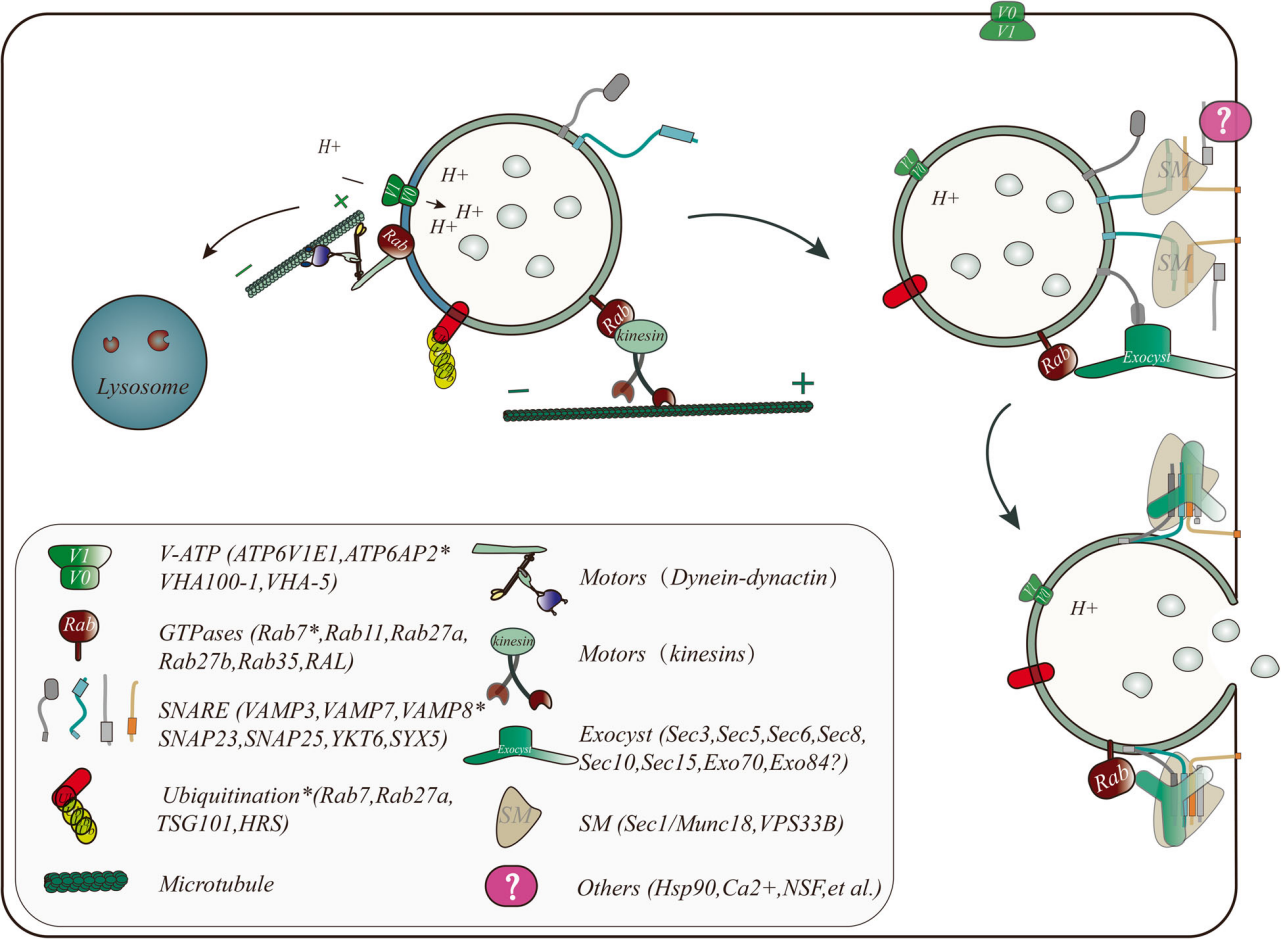
Intracellular trafficking checkpoints involved in MVB transport and fusion. Of note, as the release of exosomes requires tightly regulated steps of transport, tethering and fusion of MVBs to the plasma membrane. Moreover, MVB acidification and PTMs (mainly ubiquitination) of membrane proteins of MVBs play important roles in the regulation of MVB fate. The core factors involved in MVB trafficking are shown in the figure, where the factor labelled with ^*^ is mainly involved in MVB-targeted lysosomal degradation

MVB biogenesis may progress through one of two maturation or sorting stages, comprising direction to lysosomes where their content is degraded, reaching the cell surface where they fuse with the plasma membrane for exosome release [4, 36, 80] (Fig. 1). Therefore, a deeper understanding of the switch that determines the fate of MVBs is required, which could lead to control of the fate of MVBs. In this review, we will focus on the transport of MVBs to the plasma membrane for the release of ILVs. The ultimate fate of MVBs involves two consecutive steps: critically targeted trafficking and fusion with the biological membrane; however, the effectors involved in targeting MVBs to the lysosome or the plasma membrane are different [81–83]. Accumulating evidence has revealed the complexity of the control of MVB fate. Here, we summarize data from studies of the important factors of MVB fate. However, the different classifications have overlapping areas, indicating the complexity of MVB fate determination (Fig. 2).

#### MVB trafficking

The targeted movement and regulation of MVBs are key factors in determining their fate, which not only requires the dynamic action of molecular motors (kinesins, dynein, and actin-based myosin motors), but also the microtubule cytoskeleton, which serves as a “railroad” for trafficking [11, 84–88]. The transport of MVB-dependent molecular switches (small GTPases) along the microtubule network to its terminus, after balancing the dynamics and resistance, ultimately achieves the MVB docking and fusion with the target membrane [36, 87, 89] (Fig. 2).

By recruiting of various effector proteins, members of the Rab family of small GTPases serve as multifaceted organizers of almost all membrane trafficking processes in eukaryotic cells [32, 90–93]. Importantly, further in-depth analysis showed that small GTPases (Rab27, Rab35, Rab11, Rab7, and RAL-1) play well-established roles in MVB trafficking or docking to the plasma membrane for exosome release [51, 69, 89, 91–95]. Rab27 (Rab27 and Rab27b) and their effectors Slp4, Slac2b, and Munc13–4 play roles in MVB biogenesis and trafficking [89, 92, 96]. For example, loss of Rab27a or Rab27b function alters MVB morphology and docking to the plasma membrane, resulting in a significant drop in exosome production [92]. The Rab27a effector munc13–4 also regulates the secretion of exosomes via the Ca_2_^+^-induced Rab11-dependent MVB trafficking pathway, which influences upstream of exosome release [89, 92].

Molecular motor proteins play key roles in MVB trafficking, which including kinesin heavy chains (KIFs), the cytoplasmic dynein heavy chain, and the actin cytoskeleton and its associated myosin motors [36, 95, 97]. Generally, kinesin motors drive MVB transport from the minus-end to the plus-end (centrifugal transport), while the cytoplasmic dynein motor drives MVB transport in the opposite direction (centripetal transport) [36, 95] (Fig. 2). For example, kinesin family proteins (KIFs) and cytoplasmic dyneins are essential for outward or inward cellular prion protein (PrP^C^) + MVB trafficking, respectively [36]. Importantly, Heisler et al. confirmed that muskelin, binds to cytoplasmic dynein, is necessary for PrP^C+^ MVB inward trafficking and degradation [36]. In addition, Sinha et al. confirmed that cortactin (actin cytoskeletal regulatory protein) can bind to the branched actin nucleating Arp2/3 complex and further control both trafficking and plasma membrane docking of MVBs [84]. The difference in mechanisms is that actin-binding cortactin regulates the MVB-targeted plasma membrane, whereas the muskelin-dynein interaction controls the lysosomal targeting of PrP^C^-containing MVBs [36, 84].

However, other evidence supports the concept that PTMs of membrane proteins of MVBs play important roles in the regulation of MVB fate [33, 81, 82, 98] (Fig. 2). ISGylation (the conjugation of proteins with interferon stimulated gene 15 (ISG15)) of the MVB protein TSG101 induces its aggregation and degradation, which significantly impairs exosome secretion [81]. Similarly, Chakrabarti showed that mahogunin triggers the fusion of amphisomes and MVBs with lysosomes via the ubiquitination of TSG101, and is involved in the degradation of abnormal proteins in cells [98]. Interestingly, KIBRA (a scaffold protein in various cell processes) inhibits the proteasomal degradation of Rab27a by inhibiting the ubiquitination of Rab27a, which promotes exosome secretion [82]. Ubiquitin-specific protease 32 (USP32) can control the recycling and release of MVBs through deubiquitylation of Rab7 [99]. Similarly, Anderson et al. confirmed that phosphatidylinositol-4-phosphate 5-kinase type 1 gamma (PIPKIγi5) and sorting nexin 5 (SNX5) prevent HRS ubiquitination, which facilitates HRS sorting EGFR into MVBs and targeting to lysosomes [33]. Thus, the PTMs of MVB membrane proteins (such as TSG101, Rab27a, Rab7, and HRS) are important for the regulation of MVB fate, which may be an effective target for manipulating the fate of MVBs.

#### Fusion of MVBs with the plasma membrane

MVBs dock at the plasma membrane and ultimately complete membrane fusion to achieve exosome release [16, 100, 101]. In this review, we mainly discuss the factors that regulate MVB and plasma membrane-fusion events. Several lines of evidence support the notion that the assembly and cycling of a functional SNAREpin consisting of one R-SNARE (mostly v-SNARE) and three Q-SNAREs (mostly t-SNARE) that combine with each other to catalyze the fusion process [16, 101] (Fig. 2). Notably, the same SNARE proteins are not constitutively expressed in all cell types, which implies that each cell type may adapt a unique functional SNAREpin for its own membrane-fusion events [17, 101, 102]. The pairing of distinct SNAREs determines the specificity of the fusion, which most likely depends on the organism, cell type, or MVB subtype [16, 17, 101, 103]. It is currently believed that the process involves the following successive steps or simultaneous steps.

Numerous studies have demonstrated that Rab GTPases play active roles in SNAREpin formation. This is exemplified by Rab27a and its effectors, which, in addition to mediating vesicle-motor attachment, also control exocytic vesicle docking at the plasma membrane [90, 92, 100, 101]. The Rab27a effector interacts directly with the SM protein (sec1/munc18), which induces the docking of exocytic vesicles to the plasma membrane [90, 92]. Moreover, Rab27a silencing reduced the docking of MVBs and increased their size, which implied that the loss of Rab27a caused MVBs to fail to fuse with the plasma membrane and derives the MVBs fused to each other to become larger [1, 69, 90, 92]. In contrast, Rab27b seems to interfere with the intracellular polar distribution and targeted transport of MVBs [92]. Mammalian target of rapamycin (mTORC1) regulates exosome release through a Rab27a-dependent mechanism in HeLa cells [27]. In contrast, experiments have also shown that, in epithelial cells (ECs) and HepG2 cells, mTORC1 can promote exosome release by inducing the expression and colocalization of vesicle-associated membrane protein 3 (VAMP3) and SNAP23 [12, 94].

**Fig. 3:**
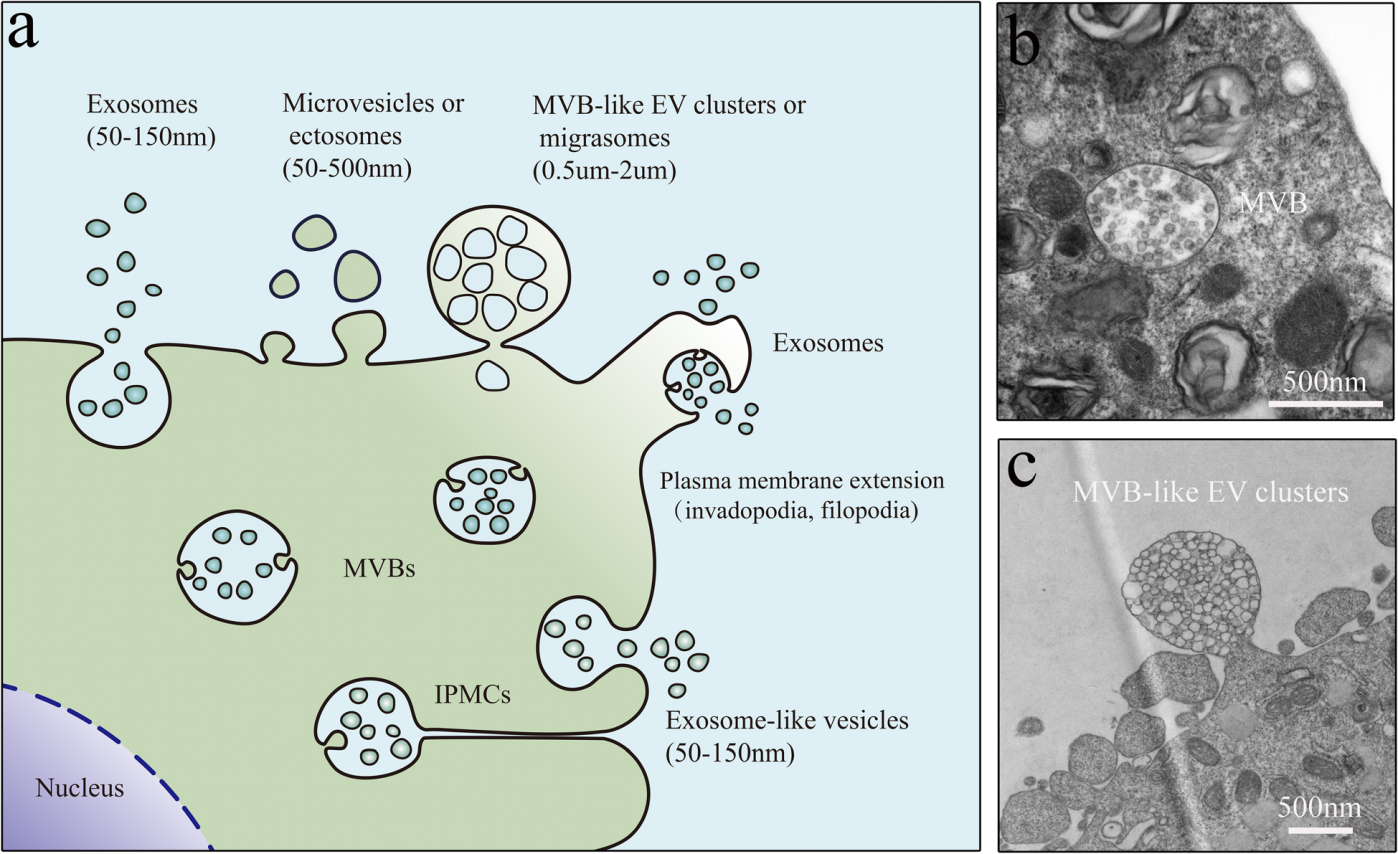
EVs secretion in eukaryotes. **a.** Multiple types of EVs originate through multivesicular endosome, plasma membrane and intracellular plasma membrane-connected compartment (IPMC) budding pathways, respectively. Importantly, exosomes are the contributors of MVBs to the total EV population(s), which are secreted during the fusion of multivesicular late endocytic compartment MVBs with the plasma membrane. Meanwhile, invadopodia (plasma membrane extensions) serve as key docking sites for exosome-containing MVBs and effectively control the quantity of exosomes secreted from cancer cells. **b and c.** Electron microscopy images of classical MVBs and MVB-like EV clusters, respectively. Images (**b** and **c**) were kindly provided by Fuhui Zhang (Department of Cell Biology, Key Laboratory of Medical Cell Biology, Ministry of Education of the PRC, China Medical University, China)

The docking and tethering of MVBs to the plasma membrane is also inseparable from the exocyst complex [100, 103] (Fig. 2). In the exocyst complex, Sec3 directly interacts with Sso1/2 to promote the initial assembly of the Sso-Sec9 t-SNARE complex and stimulate membrane fusion [100]. However, RAL1-mediated MVB plasma membrane fusion acts independently of the exocyst, and an active form of RAL-1 could derive or recruit syntaxin 5 (SYX5) aggregates on the apical plasma membrane to promote MVB fusion, thereby inducing exosome release [1]. In addition, *SYX5* silencing induced an accumulation of MVBs under the plasma membrane in mammals [1]. Importantly, actin can also induce MVB plasma membrane docking and fusion [84, 86]. Especially, invadopodia (plasma membrane extensions), specialized invasive actin structures, play key roles at docking and secretion sites for CD63-and Rab27a-positive MVBs [85], and invadopodia biogenesis and matrix-degrading activities are inextricably involved with VAMP7 and SNAP23 complexes [84]. In summary, the SNARE complex is at least partially involved in the formation of invadopodia, which indirectly affect the secretion of exosomes, suggesting that there may be a positive feedback mechanism involving the SNARE complex and invadopodia that has an important role in the secretion of tumor exosomes [84, 85] (Fig. 3a). Furthermore, pyruvate kinase type M2 and histamine can promote the fusion of MVBs with the plasma membrane via the phosphorylation of SNAP23 at serine 95 or serine 110 in tumor cells, respectively [14, 15]. Notably, the process of MVB docking and fusion in various cell types is induced by Ca_2_^+^, which may play a role in the activation of functional Rab and SNARE proteins (see above) [38, 89, 93]. Remarkably, HSP90 can also directly interact with and deform membranes via a conserved amphipathic helix, which suggests that its unique membrane-deforming function may provide the driving force for the fusion of the plasma membrane and MVBs and thus the release of exosomes [104].

The ATPase N-ethylmaleimide-sensitive factor (NSF) and its adaptor protein disassemble the SNARE complex to recycle SNARE for another round of fusion, which is a necessary step in the fusion cycle [16, 101, 103, 105]. The SM protein is involved in SNARE-dependent membrane fusion [101, 105–107]. Studies have shown that VPS33B, which contains a sec1-like domain, is a regulator of SNARE-mediated membrane fusion in arthrogryposis-renal dysfunction-cholestasis (ARC) syndrome [106]. Additionally, in hematopoietic stem cells (HSCs), VPS33B interacts with the Rab11a/Rab27a pathway to promote the secretion of exosomes [105]. The SM protein family may be involved in MVB transport by coordinating SNAREs serving multiple roles in fusion events [105, 107]. The v-ATPase and two-pore channels (TPCs), localized on the surface of endosomes and lysosomal systems, are involved in the regulation of the fate of MVBs in an acidification-dependent manner [108–111]. Moreover, these findings revealed that the intracellular vesicle pH can directly affect the final fate of intracellular vesicles, with a higher MVB pH facilitating its targeting to the plasma membrane [14, 109] (Fig. 2). In addition, the v-ATPase proton pump activity-independent v-ATPase subunit might act downstream of SNARE and participate in a late critical step of MVB-plasma fusion [111, 112]. Interestingly, in flies, depletion of VHA100–1 leads to vesicle accumulation in synaptic terminals, suggesting a defect in the release of MVBs [112]. Moreover, the abnormal function of VHA-5 (the largest subunit of the V_0_ ATPase) also led to abnormal MVB accumulation, which affected MVB docking and membrane fusion [113].

### The crosstalk between MVBs and autophagy

With the deepening of recognition of the eukaryotic endosomal membrane system, MVB biogenesis and autophagic flow also play an important role in responding to stress and maintaining cell homeostasis in pathological processes. What is the relationship between the autophagy and endocytic processes? One of the typical fates of MVBs involves autophagosomes or lysosomes in the degradation of MVB cargoes [81, 114]. Evidence has demonstrated that secretory autophagy plays an important role in unconventional protein secretion and exerts a key regulatory influence on diseases, especially tumors [114–116]. Recently, high-mobility group box 1, released by autophagic cancer-associated fibroblasts, was observed to maintain the stemness of luminal breast cancer cells [116]. Amphisomes, formed by the fusion of autophagosomes and MVBs in the rat liver, have been characterized in indirect, but not functional terms [117]. In recent years, amphisomes have garnered extensive interest. In K562 cells, Fader et al. found that induction of autophagy or overexpression of LC3 promoted the fusion of MVBs with autophagic vacuoles and inhibited exosome release [118].

However, several lines of evidence support the idea that MVB fusion with autophagosomes to form amphisomes that can also target the plasma membrane for the release of EVs [13, 37, 38, 64, 83, 119, 120]. Inhibition of PIKfyve (phosphoinositide kinase, FYVE-type zinc finger containing) using apilimod or its depletion in PC-3 cells increased the secretion of exosomes and induced secretory autophagy and observed a small population of p62-labelled electron dense structures together with CD63-containing exosomes, implying that these pathways are tightly linked [64]. This result have been caused by impaired fusion of lysosomes with MVBs or autophagosomes, which possibly increased the fusion of MVBs with autophagosomes, suggesting that cells maintain their homeostasis by secreting the contents of these vesicles [64, 119]. The release of EVs containing SNCA (alpha-synuclein) was facilitated by the inhibition of the autophagy-lysosome pathway in human neuroglioma cells, and transmission electron microscopy (TEM) confirmed that amphisomes served as novel secretory organelles in this regulation of cellular homeostasis [119, 120]. Notably, mechanical stress can induce autophagy component release via sEVs through the amphisome pathway [38]. More importantly, Jeppesen et al. confirmed that active secretion of cytosolic DNA occurs through an amphisome-dependent mechanism in DKO-1 cells [13]. In summary, these studies revealed that there is an intrinsic and complex crosslinked relationship between MVBs and autophagy. Amphisomes are formed by the fusion of MVBs with autophagosomes and not only participate in MVB degradation, but are also secreted into the microenvironment to participate in broader cell-cell communication [34, 121–123]. Previously, scholars believed that this mechanism belonged to one of the types of secretory autophagy [60, 124]. However, in recent years, several lines of evidence have supported the notion that the secretion or degradation via an amphisome-dependent mechanism might exist [125–129] (Fig. 1, Table 1). Obviously, this complex interrelationship between MVBs and autophagy requires further exploration.

**Table 1:** Secretion or degradation of cargo through an amphisome-dependent mechanism

Authors/Years	Cargoes	Cell lines	Amphisome’s judgment	Ref
**Secretion of cargo through an Amphisome-Dependent Mechanism**				
Dennis K. Jeppesen et al./2019	dsDNA and histones	DKO-1, Gli36	SIM, Colocalization (CD63、LC3)	[13]
Kaizhe Wang et al./2019	Autophagy-associated proteins	Hela, MDA-MB-231	TEM, Colocalization (CD63、LC3)	[38]
Sandra Atienzar-Aroca et al./2018	VEGFR2	ARPE-19	TEM	[125]
Georgia Minakaki et al./2018	SNCA/alpha-synuclein	Human neuroglioma cells	TEM	[120]
Elisabet Barbero-Camps et al./2018	Amyloid beta (Ab)	Neuron-rich primary cultures	–	[37]
Ying-Da Chen et al./2017	Annexin A2	Human lung epithelial cells.	Colocalization (CD63、LC3)	[126]
Nina Pettersen Hessvik et al./2016	Autophagy-associated protein (NBR1, p62, LC3, WIPI2 etc)	PC-3	TEM	[64]
**Degration of cargo through an Amphisome-Dependent Mechanism**				
Amengual J et al./2018	Apolipoprotein B100	Huh7	Colocalization (EEA1、LC3)	[127]
Jakob Mejlvang et al./2018	Autophagy receptors p62/SQSTM1, NBR1, NDP52, NCOA4	A549, BJ	Immuno-EM (p62), Colocalization (Rab5、LC3)	[63]
Guodong Wang et al./2017	Endocytic PEI-Alg NPs	Endothelial progenitor cells	TEM	[128]
Prasad Tammineni et al./2017	Association of soluble Aβ oligomers	COS7,	Colocalization (Rab7、LC3)	[129]
Ruud H. Wijdeven et al./2016	Cytosolic components	Hela, HEK 293 T	Colocalization (LC3、CD63)	[123]
Zhihua Chen et al./2016	Endocytic Ultrafine PM	Human bronchial epithelial cells	TEM	[121]
Sovan Sarka et al./2013	Cholesterol	MEF	Colocalization (Rab7、LC3)	[34]
Yusong Zhang, et al./2012	Endocytic HMGB1	HepG2	Colocalization (HMGB1、LC3)	[122]

*SIM* Structured illumination microscopy, *TEM* Transmission electron microscopy, *Immuno-EM* Immuno-electron microscop, *Colocalization* Immunofluorescence colocalization, *VEGFR2* Vascular endothelial growth factor receptor, *HMGB1* High mobility group box 1

### The contributions of MVBs to the heterogeneity of EVs

There is mentioning of the ongoing debate on the contributions of MVBs to the total EV population(s) [13, 17, 19, 69, 130] (Fig. 3). Indeed, extracellular vesicles constitute a heterogeneous population of membrane vesicles in various modes of biogenesis. Extracellular vesicle size may varies (typically 50–500 nm but as large as 10 μm), including microvesicles [131], microparticles, ectosomes [132], MVB like structures (Fig. 3a,c) (migrasomes [133], multivesicular cargo [134], and MVB-like EV clusters [135]), and arrestin­domain­containing protein 1 (ARRDC1)­mediated microvesicles (ARMMs) (similar to a subpopulation of exosomes) [136] originating from outward budding at the plasma membrane, with exosomes displaying the exosomal markers CD63, CD81, and CD9, derived from MVB-plasma membrane fusion events [1, 15, 82]. Moreover, in human monocyte-derived macrophages (MDMs), HIV-1 buds into and accumulates on the intracellular plasma membrane-connected compartments (IPMCs, also termed virus-containing compartments) [137, 138]. IPMCs are made up of complex intracellular networks of membranes, with interconnected tubular components, and channel-like connections to the cell surface, allowing them to play key roles as reservoirs of vesicle accumulation and sources for pulsatile release [137, 138]. The vesicles (similar to a subpopulation of exosomes) from IPMCs usually expressing CD81, CD9, CD53,and CD63, are also comparable to that of exosomes and sEVs [69, 137, 139]. Such similarities make the separation of virions and exosomes from virus-infected cells particularly challenging [138, 140, 141]. Taken together, we can confirm that MVB plays an important role in the composition and characteristics of extracellular vesicles, which determines the secretion and specificity of most exosomes [2, 30, 142] (Fig. 3a). Moreover, when discussing the effects of EVs derived from MVBs on disease, we must strictly ensure that the exosomes are of multivesicular endosomal origin to avoid exaggerating the role of MVBs in pathophysiology.

### MVBs in tumor progression

MVBs are involved in promoting virtually all aspects of cancer progression, including tumor expansion, immune responses, and drug resistance [2, 28, 30, 71, 143]. Studies have shown that MVBs, as important mediators of many aspects of cellular homeostasis, undergo significant changes in adapting to various stress conditions that enable cancer cells to maintain its homeostasis [3, 24, 40]. Additionally, tumor cells are generally exposed to high levels of stress, such as starvation, hypoxia, chemotherapy drugs, pH, and various inflammatory factors [131, 144, 145]. When cancer cells need to escape unfavourable environments, on the one hand, they can release special exosomal cargo, such as nucleic acids, signalling proteins, and metabolites, and thus mediate the exchange of information between the tumor and its microenvironment [39, 142, 144, 146] 32,446,697. On the other hand, metabolic waste and catabolized toxic substances are released via an endosomal-lysosomal mechanism, which provides recycled raw materials [30, 36]. As highlighted in the previous sections, we discuss the role of MVBs in material balance via the endosomal-lysosomal system and the release of exosomes from MVBs containing selective cargoes that participate in intercellular substance regulation, thereby producing a comprehensive perspective on the multiple roles that MVBs play in tumor progression and metastasis (Fig. 1).

#### MVBs and metastasis in cancer

Mounting evidence confirms that, in tumors, the key proteins involved in MVB formation, transport, fusion, and other steps are abnormally expressed, suggesting that dynamic changes to MVBs play vital roles [2, 12, 147]. For example, the abnormal expression of STX1A and VAMP2 promotes the progression and invasion of cancer cells, transforming cells into high-grade tumors in bladder cancer [147]. Valcz et al. identified the most aberrantly expressed MVB markers and revealed the transition of diffuse ALIX signals into a MVB-like pattern during the adenoma-carcinoma sequence, which may provide clues to revealing the interactions between cancer and the surrounding microenvironment, particularly those involving the regulation of tumor growth and metastatic invasion [148]. Moreover, the formation of invadopodia (see above) in many types of cancer drives cell invasion, depending on enhanced MVB docking and fusion with the plasma membrane [85, 102, 149]. Therefore, we believe that invadopodia formation is the first step in tumor cell invasion and metastasis, in which MVB-mediated transport of MMPs to degrade the extracellular matrix is an important step [85, 89, 102, 149]. A recent study revealed that VAMP3-dependent secretion of MT1-MMP enhances the degradation of the extracellular matrix, and induces cancer cell invasion [102]. Although the concept of invadopodia was not mentioned, a study showed that MVB-mediated targeting of MMPs to plasma membrane plays an important role in initiating tumor cell invasion and metastasis [85, 102] (Fig. 3a). Importantly, MVBs can selectively load active substances (see above) to exert protumorigenic effects on stromal cells via the paracrine or autocrine signalling [2, 29, 39, 146] (Fig. 1). For example, the secretion of extracellular vesicle-packaged HIF-1α-stabilizing lncRNA by tumor-associated macrophages inhibits the hydroxylation and degradation of HIF-1α by blocking the interaction of prolyl hydroxylase domain 2 and HIF-1α in breast cancer cells, facilitating their aerobic glycolysis and chemoresistance [39]. Hepatoma cell-derived exosomal miR-103 increases vascular permeability and facilitates metastasis by targeting multiple endothelial junction proteins [146].

#### MVBs and immunity in cancer

Immunotherapy has revolutionized cancer therapy, among which programmed death ligand 1 (PD-L1) has been proved to be an effective target for inhibiting tumor growth [30]. Strikingly, several studies identified that MVBs play key roles in the cell-surface expression and exosomal packaging of PD-L1 [30]. ALIX regulates the immunosuppressive properties in basal-like breast cancer (BLBC) cells by enhancing PD-L1 sorting onto ILVs and its release into the microenvironment to deplete PD-L1 surface presentation [30]. PD-L1 localized to the limiting membrane and ILVs of CD63-positive MVBs in HCC1954 cells can be degraded by lysosomes or be returned to the plasma membrane [30]. Importantly, PD-L1 on EVs may be important mediators of immunosuppression for glioblastoma and support the potential of EVs as biomarkers in glioblastoma patients [150]. Obviously, the fate of MVBs in mediating PD-L1 significantly affects the immune escape and treatment of tumors [30, 142]. Moreover, many studies have confirmed that exosomal PD-L1 plays an important role in mediating tumor metastasis, immune escape, and immunotherapy. Importantly, studies on glioblastoma, metastatic melanoma [151], breast cancer [152], and prostate cancer [142] have confirmed that exosomal PD-L1 can mediate resistance to immunotherapy by directly binding to an anti-PD-L1 antibody [153]. Remarkably, some scholars have discovered that the combination of an exosomal PD-L1 blockade and anti-PD-L1 antibodies has the potential to suppress tumor growth and improve antitumour response in the clinic [142, 154]. Importantly, many receptors or membrane proteins, such as vascular endothelial growth factor receptor (VEGFR) and growth factor receptor (GFR) have the potential to undergo a similar fate as PD-L1 in tumorigenesis [28, 29, 102, 142]. Manipulating the fate of MVBs is an important prospect for improved cancer treatment.

#### MVBs and drug resistance in cancer

Cells have the ability to antagonize drug effects through various mechanisms, and MVBs play an important role in the mechanism of cell-acquired resistance [155–157]. In particular, drug resistance is an important factor in the poor prognosis of patients with cancer; thus, the role of MVBs in drug resistance is essential [155, 158]. In recent years, emerging evidence has suggested that MVBs selectively load exosomes containing active proteins, RNAs, and other substances to transfer drug resistance between cells [40, 155, 158]. For example, Xu et al. found that PSMA3 (encodes proteasome subunit α7) and lncPSMA3-AS1 can be packaged into exosomes to transfer proteasome inhibitor resistance from mesenchymal stem cells to multiple myeloma cells [158]. Macrophage-derived exosomes are conveyers of antagomirs, which induce gemcitabine resistance in pancreatic adenocarcinoma [40]. Moreover, MVBs can control membrane receptor recycling or degradation via the lysosomal pathway, which can alter the toxic responses of drugs on target cells [28]. Qu et al. revealed that HCC cell-derived exosomes induce sorafenib resistance [156]. Whereas, Ardelt et al. demonstrated that the MVB-mediated endocytosis of VEGFR and GFR attenuated the HCC cell response to sorafenib [28] (Fig. 1). The investigators demonstrated that arresting MVB intracellular trafficking using cyclin-dependent kinase 5 inhibitors, led to the intracellular accumulation of various cargoes that likely affected the extent and quality of VEGFR and GFR signal activation, significantly improving the efficacy of sorafenib [28]. Similarly, Dutta et al. confirmed that neuropilin 2 (NRP2) depletion impaired the endocytic transport of cell surface EGFR, which blocked the functionally active EGFR in MVBs, thereby triggering aberrant ERK activation and cell death [29].

## Conclusions

This review summarizes recent progress in the research into MVBs. Initially, the mechanisms of ILV formation were discussed. Second, the sorting mechanisms of different cargoes were analyzed, with special emphasis on the possible mechanisms of sorting cargoes such as cytoplasmic proteins, RNAs, and lipids into MVBs.

Clearly, different cargoes are loaded into MVBs via a variety of methods, and the more distant cytoplasmic cargoes are not randomly targeted to MVBs, which suggests the presence of a special recruitment mechanism. Current research confirms that RNA is recruited through the facilitation of multiple RBPs (e.g., RISC). However, the only known mechanism by which cytoplasmic proteins are sorted into the lumen of MVBs involve the eMI and NVT pathways.

We also highlighted the importance the fate of mature MVBs in stimulating tumor growth, metastasis and drug resistance, and the manipulation of MVBs to treat tumors is gradually becoming a possibility. Considering on the complexity of the endosomal system, we have generated a reasonable summary of current research and speculated on the factors that might regulate the fate of MVBs. Although many mechanisms for MVB regulation are summarized, there are many details that remain to be determined, including whether there is a difference in the dominant regulatory mechanisms of MVBs in different tumors and stress situations. How can specifically manipulate the fate of MVBs to improve cancer diagnosis and therapy? Is it possible to predict tumor occurrence through early changes in the shape and content of MVBs? Consequently, exploring the exact mechanisms of MVB fate regulation in different diseases and establishing a comprehensive knowledge of MVBs are increasingly important.

Cells exhibit distinct classes of MVBs and EVs that are generated under different microenvironmental stresses. For example, under hypoxic conditions, tumour cells show changes in morphology, distribution, and accumulation cargo of MVBs and are accompanied by significant differences in the number, morphology, and cargoes of extracellular vesicles. Importantly, these changes indicate that, in different cellular contexts, the characteristics of MVBs and EVs undergo dynamic changes, creating great challenges and opportunities for understanding and treating diseases. In future research, we should pay more attention to the use of complementary methods of analysis and more accurate biomarkers to more accurately distinguish between different MVB subgroups and heterogeneous EVs, which would be beneficial for exploring whether there is a ubiquitous crosstalk mechanism involved in various MVB functions. At the same time, we also highlight the importance of MVBs in different aspects of cancer progression and metastasis for developing novel treatment strategies.

## Data Availability

Other datasets analyzed during the current study are available from the corresponding author on reasonable request.

## Abbreviations

MVBs: Multivesicular bodies
EVs: Extracellular vesicles
SNAREs: Synaptosomal-associated proteins
ESCRT: Endosomal sorting complex required for transport
VPS4: Vacuolar protein sorting gene 4
MLKL: Phosphorylated mixed lineage kinase domain like pseudokinase
HRS: Hepatocyte growth factor-regulated tyrosine kinase substrate
NSF: N-ethylmaleimide-sensitive factor
PIPKIγi5: Phosphatidylinositol-4-phosphate 5-kinase type 1 gamma
TSG101: Tumor suppressor gene 101 protein
PTMs: Post-translational modifications
PD-L1: Programmed death ligand 1
RBPs: RNA-binding proteins
hnRNPA2B1: Heterogeneous nuclear ribonucleoprotein A2B1
VAMP: Vesicle-associated membrane protein
VEGFR: Vascular endothelial growth factor receptor
